# Strengthening the integration of primary care in pandemic response plans: a qualitative interview study of Canadian family physicians

**DOI:** 10.3399/BJGP.2022.0350

**Published:** 2023-03-21

**Authors:** Maria Mathews, Dana Ryan, Lindsay Hedden, Julia Lukewich, Emily G Marshall, Richard Buote, Leslie Meredith, Lauren R Moritz, Sarah Spencer, Judith B Brown, Paul S Gill, Bridget L Ryan, Stephen J Wetmore

**Affiliations:** Department of Family Medicine, Schulich School of Medicine & Dentistry, Western University, London, ON, Canada.; Department of Family Medicine, Schulich School of Medicine & Dentistry, Western University, London, ON, Canada.; Faculty of Health Sciences, Simon Fraser University, Burnaby, BC, Canada.; Faculty of Nursing, Memorial University, St John’s, NL, Canada.; Department of Family Medicine Primary Care Research Unit, Dalhousie University, Halifax, NS, Canada.; Department of Family Medicine Primary Care Research Unit, Dalhousie University, Halifax, NS, Canada.; Department of Family Medicine, Schulich School of Medicine & Dentistry, Western University, London, ON, Canada.; Department of Family Medicine Primary Care Research Unit, Dalhousie University, Halifax, NS, Canada.; Faculty of Health Sciences, Simon Fraser University, Burnaby, BC, Canada.; Department of Family Medicine, Schulich School of Medicine & Dentistry, Western University, London, ON, Canada.; Department of Family and Community Medicine, Temerty Faculty of Medicine, University of Toronto, Toronto, Canada; professor, Department of Family Medicine, Schulich School of Medicine & Dentistry, Western University, London, ON, Canada.; Department of Family Medicine and Department of Epidemiology and Biostatistics, Schulich School of Medicine & Dentistry, Western University, London, ON, Canada.; Department of Family Medicine, Schulich School of Medicine & Dentistry, Western University, London, ON, Canada.

**Keywords:** COVID-19, disease outbreaks, family physician, pandemics, primary care, qualitative research

## Abstract

**Background:**

As the first point of contact in health care, primary care providers play an integral role in pandemic response. Despite this, primary care has been overlooked in previous pandemic plans, with a lack of emphasis on ways in which the unique characteristics of family practice could be leveraged to create a more effective response.

**Aim:**

To explore family physicians’ perceptions of the integration of primary care in the COVID-19 pandemic response.

**Design and setting:**

Descriptive qualitative approach examining family physician roles during the COVID-19 pandemic across four regions in Canada.

**Method:**

Semi-structured qualitative interviews were conducted with family physicians and participants were asked about their roles during each pandemic stage, as well as facilitators and barriers they experienced in performing these roles. Interviews were transcribed and a thematic analysis approach was employed to develop a unified coding template across the four regions and identify recurring themes.

**Results:**

In total, 68 family physicians completed interviews. Four priorities for integrating primary care in future pandemic planning were identified: 1) improve communication with family physicians; 2) prioritise community-based primary care; 3) leverage the longitudinal relationship between patients and family physicians; and 4) preserve primary care workforce capacity. Across all regions, family physicians felt that primary care was not well incorporated into the COVID-19 pandemic response.

**Conclusion:**

Future pandemic plans require greater integration of primary care to ensure the delivery of an effective and coordinated pandemic response. Strengthening pandemic preparedness requires a broader reconsideration and better understanding of the central role of primary care in health system functioning.

## INTRODUCTION

Primary care providers’ unique attributes call for their clear and well-considered integration in pandemic response plans. Primary care is the first point of contact in a community-based outbreak and family physicians deal with new diseases when there is little information about the nature of the disease or how it spreads.^1^ However, in the Canadian federal and provincial influenza pandemic plans published prior to the COVID-19 pandemic,^2^^–^^10^ there are few references to primary care. These documents broadly describe family physicians’ involvement in surveillance, screening, and testing, and emphasise infection prevention and control in physicians’ practices. One notable exception is Ontario’s influenza pandemic plan, which emphasises the need to maintain the availability of primary care services during a pandemic and raises the possibility of introducing new funding models to increase capacity to see more patients, including fee codes for telephone visits with vulnerable populations.^8^ Similarly, pandemic planning documents published by the World Health Organization prior to the COVID-19 pandemic make few specific references to primary care providers,^11^^,^^12^ while documents published after the pandemic highlight the importance of the primary care sector.^13^

This study explored Canadian family physicians’ perceptions of the integration of primary care into a pandemic response during the COVID-19 pandemic. The federal government in Canada has responsibility for public health guidance, while provinces have responsibility for the organisation and delivery of health care, including responses to the COVID-19 pandemic. Day-to-day management and operations are coordinated at a regional level. Primary care physicians are generally family physicians who are largely independent, small businesses owners, or subcontractors. Family physicians have traditionally been paid on a fee-for-service basis by the provincial public health insurance programme, but funding reforms in the last two decades have introduced new forms of payment such as lump sum per patient (capitation), as well as performance-based bonuses for targeted services. In addition, provinces have introduced regional networks or formalised connections to hospitals to enhance the integration of primary care with regional organisations, but membership usually remains voluntary. Reforms to primary care organisation, remuneration policies, and COVID-19 responses have varied across provinces.

**Table:** How this fits in

Previous pandemic plans have largely overlooked the important role of primary care in a pandemic response. The COVID-19 pandemic presents a novel opportunity to examine the key roles family physicians play during a pandemic and sheds light on existing barriers and supports. Findings from this study highlighted the need for greater incorporation of primary care in the development of strengthened pandemic plans.

Enhancing collaboration between primary care and other health system sectors has been shown to be integral to an effective pandemic response in international comparisons.^13^^,^^14^ This study identifies how the unique characteristics of family practice could be leveraged in a pandemic response, informs the development of pandemic preparedness, and is part of a larger project examining the roles and activities performed by family physicians during various stages of the pandemic response.

## METHOD

Using a descriptive qualitative approach, this study conducted semi-structured qualitative interviews to examine family physician roles during the COVID-19 pandemic across four regions in the provinces of British Columbia, Newfoundland and Labrador, Nova Scotia, and Ontario. The authors’ previously published protocol described the regions and the rationale for their selection.^15^ In each region, the authors interviewed family physicians between October 2020 and June 2021 using a semi-structured interview guide. By June 2021, Canada had gone through a series of phased closures and re-openings and had started vaccination of the general population,^16^ but had not experienced an acute care crisis to the degree seen in some areas of the US or parts of Europe. The authors recruited family physicians along a wide range of characteristics (having an academic appointment, genders, primary care funding and practice models, team involvements, hospital affiliations, practice settings, and community size) using maximum variation sampling until saturation was reached.^17^^,^^18^

Study-eligible family physicians were clinically active or eligible to be clinically active in their region in 2020. This study excluded physicians who were still in residency training, with temporary licences, or who held exclusively academic, research, or administrative roles. Across regions, research assistants recruited family physicians by emailing study invitations to individuals on faculty lists, practice directories (for example, family health teams and community health centres), privileging lists, and provincial medical regulatory authorities’ physician search portals. The authors also included recruitment notices in professional organisations’ newsletters, social media posts, and, where permitted, used snowball sampling.

During the interview, the authors asked about the various pandemic-related roles family physicians performed over different stages of the pandemic and the facilitators and barriers they experienced in performing these roles. The authors also asked about potential roles that family physicians could have filled, as well as questions about their background and practice characteristics. This study adapted interview questions to account for differences in physician roles and broader health system contexts in each region. Depending on participant preference, interviews were conducted by video (Zoom) or telephone and were audiorecorded and transcribed verbatim.

Using an inductive thematic analysis approach, the authors developed a coding template from the interview transcripts and interviewers’ field notes. In each region, at least two members of the research team independently read two to three transcripts to identify key words and codes, which were organised into a preliminary coding scheme and updated to incorporate additional codes from subsequent transcripts. Regional teams then met to compare and refine code descriptions, and develop a harmonised template with uniform code labels and descriptions. Teams used the unified coding template and NVivo (version 12) to code all transcripts and field notes across the four regions. The authors resolved disagreement in coding through discussion and consensus. The authors summarised participant demographic and practice characteristic data using descriptive statistics.

To enhance the rigour of the study,^17^^–^^19^ the authors pre-tested interview questions, used experienced interviewers, verified meaning with the participants during interviews, and kept a detailed audit trail. The authors searched for negative cases and used thick description and illustrative quotes.^20^ The authors drew on the expertise of their interdisciplinary team, which included family physicians and public health experts, who reviewed initial analyses.

### Positionality.

The authors were primary care researchers with training in health administration, epidemiology, social work, anthropology, nursing, public health, and family medicine, including some family physicians directly involved in pandemic response, including those in leadership positions. Through discussion of node reports and manuscript drafts, the authors arrived at a description and interpretation of findings that balanced their individual views and reflected the data (quotations) gathered from study participants. The broader research team included family physicians, public health officials, health care administrators, and policymakers who confirmed manuscript findings resonated with their own experiences.

## RESULTS

In total, 68 family physicians were interviewed (15 from British Columbia, 12 from Newfoundland and Labrador, 21 from Nova Scotia, and 20 from Ontario). The interviews lasted an average of 58 minutes. The participants had been in practice for an average of 16.9 years (range 1 to 38 years), and consisted of 41 women, 27 men, and 20 rural physicians (Table 1). The majority (*n* = 46) were paid by alternate payment plans (not by fee-for-service; for example, global funding, capitation, and salary) and had hospital privileges (*n* = 49).

**Table 1.** Characteristics of study participants by province

**Characteristic**	**Ontario, *n* = 20, *n* (%)**	**Nova Scotia, *n* = 21, *n* (%)**	**British Columbia, *n* = 15, *n* (%)**	**Newfoundland and Labrador, *n* = 12, *n* (%)**	**Total, *N* = 68, *n* (%)**
**Gender^a^**					
Men	10 (50)	9 (42.9)	4 (26.7)	4 (33.3)	27 (39.7)
Women	10 (50)	12 (57.1)	11 (73.3)	8 (66.7)	41 (60.3)

**Practice type**					
Fee-for-service	4 (20)	7 (33.3)	6 (40)	5 (41.7)	22 (32.4)
Alternative payment plan^b^	16 (80)	14 (66.7)	9 (60)	7 (58.3)	46 (67.6)

**Hospital privileges/affiliation**					
No	15 (75)	6 (28.6)	3 (20)	5 (41.7)	29 (42.6)
Yes	5 (25)	15 (71.4)	12 (80)	7 (58.3)	39 (57.4)

**Community size^c^**					
Rural	9 (45)	8 (38.1)	0	3 (25)	20 (29.4)
Small urban	1 (5)	0 (0)	0	0 (0)	1 (1.5)
Urban	8 (40)	13 (61.9)	15 (100)	8 (66.7)	44 (64.7)
Mix	2 (10)	0 (0)	0	1 (8.3)	3 (4.4)

**Years in practice, mean**	18.7	15.4	16.9	16.3	16.9

a *Gender was asked as an open-ended question.*

b *Alternate payment includes all non-fee-for-service or enhanced fee-for-service payment types.*

c *Rural*<*10 000 population; small urban = 10 000 99 999 population; and urban ≥100 000.*

The key themes described family physician perceptions of existing pandemic plans and identify four priorities for integrating primary care in future pandemic planning: 1) improve communication with family physicians; 2) prioritise community-based primary care; 3) leverage the longitudinal relationship between patients and family physicians; and 4) preserve primary care workforce capacity.

Across all four regions, family physicians reported a lack of primary care involvement in pandemic planning and the initial pandemic response. A participant from British Columbia felt that family physicians and their role in pandemic response had been overlooked:

*‘With respect to the pandemic planning, inexplicably, family physicians were completely excluded from the process of pandemic planning … ’*

(British Columbia [BC]10)

A family physician in Ontario echoed a similar sentiment, noting the need for a plan outlining roles and responsibilities:

*‘It would have been great to get some kind of top-down approach on direction, on how to coordinate the system*.*’*

(Ontario [ON]07)

Family physicians were disappointed in the gaps in guidance for primary care, and the lack of shared understanding of the roles of public health and primary care during the pandemic:

*‘… we expected Public Health to … give us a pre-made pandemic plan. We* […] *always assumed that this was directly in the ballpark of Public Health and that there were documents that existed … like we have code orange documents and other disaster planning documents at our hospital. Public Health subsequently has sort of told us that they see their role more as managing contact tracing and things in the community and they feel they have no jurisdiction over how we run our practice, so it was left more up to us … to determine how we were going to implement provincial policies.’*

(ON04)

Participants identified four priorities for better integration of primary care into future pandemic plans. These priorities address the challenges many experienced during the COVID-19 pandemic.

### Improve communication with family physicians.

Participants believed that there was insufficient communication between health system and public health and primary care providers. In Newfoundland and Labrador, a family physician described the collective frustration with regional updates:

*‘There was no formal communication between* […] *the regional health authority and the community-based physicians until well into it, when we demanded meetings to have updates on what* [the regional health authority] *was doing.’*

(Newfoundland and and Labrador [NL]11)

The physician noted that basic infrastructure for communicating with community-based family physicians was not in place prior to the pandemic:

*‘We were told that we couldn’t receive the emails unless we had an* [regional health authority] *account.’*

(NL11)

Family physicians also felt that the existing guidance was not applicable or well-tailored to the family practice setting. For example, directives for infection prevention and control practices were not targeted to family physician practices:

*‘*… *people in general practice felt not as guided by the health authority as other parts of the health system … There was lots of guidance about how you should behave in hospitals, or how clinics were going to work in hospitals … But there was no guidance about how should I redesign my waiting room if I’m in a general practice office … ’*

(Nova Scotia [NS]01)

Similarly, family physicians in Nova Scotia perceived that they were left on their own to sort out arrangements:

*‘*… *there was such ambiguity initially that everybody was a little left to fend for themselves*.*’*

(NS19)

While family physicians were called on to enact or facilitate pandemic response, they felt they had insufficient time to make sense of new information before explaining it to patients:

*‘We* [family physicians] *would like to be informed … one day before* [new guidance] *gets out to the public*.*’*

(BC01)

In Newfoundland and Labrador, a family physician recounted how she learned about the transition to telephone appointments from her patients rather than from an official source:

*‘I found the biggest gap* [in regards to communication] *was from the government to us … I was often the last to know about something … Like, I remember being told by someone that, “Oh, you guys are doing phone calls now,” … but I wasn’t given information as to what billing code that was, or what I had to record … it would have been nice to know that before the general public.’*

(NL10)

Participants also expressed frustration about the lack of notice when new screening tools for school-aged children were announced:

*‘The school screening tool,* [the government] *put it out publicly and then we have parents calling us and we haven’t even had a chance to digest it ourselves or read it or understand what advice to give.’*

(ON01)

While family physicians were routinely expected to translate public health directives to their patients, they were often left without adequate preparation time or information.

### Prioritise community-based primary care.

Many participants expressed how the initial pandemic response plans did not account for the contribution of family physicians to the daily management of health services within the healthcare system:

*‘I just felt like they forgot that primary care is really where a lot of … patient care happens.’*

(NS15)

Family physicians noted that a functioning primary care system was instrumental to ensuring that the broader health system does not become overwhelmed:

*‘The whole model should have realised that primary care was … the only thing that was going to prevent the system from being overrun.’*

(NL11)

They highlighted the focus of pandemic plans on hospitals, even though the bulk of cases did not require tertiary care:

*‘It was just shocking to me how overlooked primary care was in the planning for a disease that … had a 6% hospitalisation rate, so the other 94% of patients with symptoms, or questions even, or contacts of people who had symptoms, are outside of the hospital. But all of our attention was put on the hospital.’*

(ON01)

Primary care providers received little guidance on managing COVID-19 cases in their practice even though many patients may have been expected to seek care for COVID-19 from their family physician:

*‘Who do* [people with flu-like illnesses] *see when they’re sick? Us. Especially mild, moderate illnesses that don’t need an* [emergency room visit] *and don’t need an ICU* [intensive care unit]*? They come and see us*.*’*

(NS15)

In addition to caring for patients with COVID-19, family physicians continued to provide routine care, especially for patients with chronic diseases, to ensure these patients did not add to the pressure on tertiary care services that may already be strained due to COVID-19:

*‘A well-functioning primary care system can help a tertiary system take care of ongoing issues related to COVID. If patients who have diabetes are not taken care of and they have cardiac events, then the acute care system can’t work to serve them. So, the reason why family doctors should be recognised in a future pandemic, it’s because we have a role in supporting the tertiary care system along the way.’*

(BC09)

### Leverage the longitudinal relationship between patients and family physicians.

The longitudinal relationship between patients and family physicians was largely under-utilised in the existing pandemic plans:

*‘*[I] *didn’t feel as though we were included in any of the planning in any way in which we could leverage our longitudinal care and relationships with patients to either keep them out of emergency departments, keep them out of walk-in clinics, to keep them informed.’*

(BC01)

Family physicians can take proactive measures to prevent health deterioration in their complex patients. For example, in one Ontario clinic, a family physician reached out to frail patients:

*‘The longitudinal relationship between family physician and patient also means that the family physician has an appreciation for the patients in a holistic sense and can anticipate care needs. The role of primary care is to really have that, that upstream lens, right? Of not waiting for health to destabilise … like we knew who was frail … So, we could instantly reach out and support the frail persons.’*

(ON03)

Their long history of caring for patients can ease rapid transitions in the delivery of care (such as the adoption of phone visits):

*‘For some people with a mix of the in-person and the telephone calls, you can do quite a lot … the biggest thing is when you’ve got people who know you. So, they’ve been our patients for 6 years and they know my voice on the phone.’*

(NL06)

Participants explained that there is a high level of trust between patients and their regular doctor, *‘People choose their family doctor … It’s a relationship built, based on trust’* (BC10), and that this trustworthiness made family physicians an influential and credible source for disseminating information about COVID-19 and related public health measures:

*‘One of the roles that many family physicians did play, was to be a source of reputable and informative scientifically-based medical information. So, we did videos for our community television … on how to properly hand wash … about why COVID is different, why it’s scary, why masks are important. And people found that very, very helpful ... it was coming from somebody they trusted … We think that we probably had stronger public health measures with regards to masks and social distancing because people heard it from us and not from another face that they really didn’t know or trust.’*

(NS05)

Trust in family physicians was particularly important in reaching communities that experience marginalisation. A physician working with patients who use substances or are experiencing homelessness stated:

*‘So there were all these rumours all the time, people were on high alert and they were looking to us to sort of demystify what was going on with COVID*.*’*

(BC13)

### Preserve primary care workforce capacity.

Family physicians are generalists with many skills, which make them the ‘go-to’ physician for filling in pandemic-related staffing gaps in hospitals, emergency rooms, long-term care facilities, and public health operations. One physician described the multifaceted utility of family physicians:

*‘We are such a … Swiss army knife, right? We’ve got so many tools … There’s a lot of places we could go to help.’*

(NS02)

The implicit assumption in existing hospital-centric pandemic plans is that family physicians will provide surge capacity as well as take on roles in assessment centres, field hospitals, mobile health units, and vaccination centres. However, family physicians noted that keeping primary care practices open and operating must be prioritised to realise the benefits of longitudinal patient relationships, preventative care, and gatekeeping to other parts of the healthcare system. Pandemic plans that require primary care physicians to redeploy must also include plans to back-fill the gaps created in the primary care system:

*‘… if you redeploy people, do they also still have some mandated time to provide primary care for their complex patients? So, if I’m working in* [the emergency department], *do I still get to run a half-day clinic a week or a clinic a week to address the needs of those patients? Or do my coworkers … can they pick up my slack?’*

(NS22)

Family physicians also noted the need for pandemic plans to create priorities for family physicians:

*‘*… *I’ve been hearing a lot of conflicting things … “we want you to be ramping back up your cancer screening” and “we want you to be seeing more people in person” and “we want you to work in an assessment centre” and “we want you to start swabbing in your office”. Well, I can’t do all of those things. So, the system needs to prioritise what they would like us to be doing …* [but] *somebody has to be continuing to look after people’s chronic and acute illnesses during a pandemic*.*’*

(ON01)

## DISCUSSION

### Summary

By exploring the experiences of family physicians in four Canadian jurisdictions, this study identified four priorities for improving the integration of primary care into pandemic response, which highlighted the unique attributes of primary care. As the usual first contact between patients and the healthcare system, and ideally centring on an ongoing relationship, family physicians are a trusted source of information for patients.^21^^–^^23^ However, family physicians need to be informed of the broader pandemic plan, their role in it, and have sufficient time and guidance to interpret, apply, and disseminate public health directives to their patients. Moreover, primary care is whole-person care provided across the patient’s life course that includes health promotion and education, routine screening and preventative services, diagnosis and management of acute and chronic conditions, and end-of-life care.^21^^,^^22^ Ensuring that family physicians have the resources to continue to deliver these types of care alleviates downstream demand in the health system.^24^^,^^25^

Given their broad skills and scope of practice, family physicians are expected to take on many roles in a pandemic;^2^^,^^16^ however, primary care providers should be redeployed judiciously, taking into account the skills required to fulfil pandemic roles while also preserving primary care capacity to continue fulfilling the roles for which family physicians are uniquely qualified (acting as a trusted resource, caring for pandemic cases in the community, continued preventative care, and chronic disease management). Given the regional variation in local needs and the organisation and delivery of care, family physicians need to be involved in leadership positions in pandemic planning and redeployment decisions.^26^

Pandemic plans need to ensure that the infrastructure, resources, and training needed to carry out pandemic roles are available to family physicians. Most family practices in Canada operate as private businesses and, therefore, communicating and coordinating with individual practices may be difficult, especially if family physicians do not have formal linkages with regional or institutional networks or have regional governance structures.^27^ Communications with family physicians about region-specific pandemic-related operational matters were facilitated if email lists were in place (for example, if family physicians held academic appointments, had hospital privileges [were permitted to admit and see patients in hospital or the emergency department and refer patients for hospital-based diagnostic services], or belonged to a primary care network). As private businesses, family practices may neither stockpile nor have had access to the amount and quality of personal protective equipment needed to provide in-person care, especially when demand for personal protective equipment skyrocketed and supply lines were disrupted.^28^^–^^30^ Poor access to adequate personal protective equipment during the COVID-19 pandemic contributed to an erosion of trust in clinical judgement and sense of workplace safety.^30^ Additionally, while provincial pandemic plans had identified the need to implement fee codes for virtual care,^3^^,^^8^ many primary care providers had not been trained to provide virtual visits and infrastructure was new or had not yet been implemented.^31^ These findings suggest that pandemic plans prior to COVID-19 did not account for the organisation of primary care in Canada.

### Strengths and limitations

Pandemic experiences and primary care systems in other regions may differ. Some physician perspectives (such as solo practitioners) may not be fully reflected in this study’s data. This study’s data, collected between October 2020 and June 2021, may not reflect pandemic response during later stages of the pandemic. Interview data may be subject to recall bias^32^ and social desirability bias.^33^

### Comparison with existing literature

The findings show that family physicians play important roles in realising the public health goals in a pandemic plan. In Canada, public health and primary care are viewed as separate health system sectors.^34^ While awareness of the overlap in goals of the two sectors is not new, previous efforts were largely focused on better integration of a population health perspective into the health promotion and prevention services delivered in primary care.^35^^–^^37^ Studies conducted prior to the pandemic have identified organisational^35^ and system factors^37^ that promote collaboration between the two sectors but did not examine collaboration in the context of a pandemic response.

The findings of this study echo reports from other countries. Primary care providers in high-income countries felt unprepared in early stages of the pandemic,^14^ especially in light of evolving information^38^^,^^39^ and guidelines that focused on secondary settings.^40^^–^^42^ Community-based (or private) primary care physicians in Greece^38^ and the US^14^ reported poorer access to information than physicians with stronger connections to healthcare organisations. Primary care physicians in Europe (including the UK) and the US reported a reduction in their ability to provide routine primary care, rapid adoption of virtual care, and limited anticipation of the need to sustain primary care for an extended period during the pandemic.^26^^,^^40^^,^^42^^–^^44^ Studies have also noted that the pandemic has highlighted the importance of primary care to patient health,^14^^,^^38^^,^^39^^,^^45^ and primary care physicians from the US have similarly pointed to the need to leverage the unique patient–physician relationship in pandemic response.^45^

### Implications for practice and research

The findings suggest that pandemic preparedness must articulate the roles family physicians are expected to perform and incorporate the resources needed to support the provision of ongoing primary care (such as access to appropriate personal protective equipment, billing codes to facilitate virtual care, guidelines to enhance infection protection and control in primary care settings, and communication infrastructure in the response plan). The findings also call for research, in Canada and elsewhere, to evaluate how the organisation of primary care and associated reforms, and the integration of primary care and public health, have helped or hindered a coordinated pandemic response.

Family physicians suggested four priorities to strengthen pandemic preparedness: improving communication with family physicians; prioritising community-based primary care; leveraging the longitudinal relationship between patients and family physicians; and preserving primary care workforce capacity. Strengthening the integration of primary care in pandemic planning requires a reorientation of pandemic preparedness to community-based care and a reconsideration and better understanding of the role of primary care in health system functioning.
